# Sliding Wear Behavior of High-Temperature Vacuum-Brazed WC-Co-NiP Functional Composite Coatings

**DOI:** 10.3390/ma15010088

**Published:** 2021-12-23

**Authors:** Roxana Muntean, Dragoș-Toader Pascal, Norbert Kazamer, Gabriela Mărginean, Viorel-Aurel Șerban

**Affiliations:** 1 Department of Materials and Manufacturing Engineering, University Politehnica of Timișoara, Piața Victoriei 2, 300006 Timisoara, Romania; roxana.muntean@upt.ro (R.M.); viorel.serban@upt.ro (V.-A.Ș.); 2 Westphalian Energy Institute, Westphalian University of Applied Sciences Gelsenkirchen Bocholt Recklinghausen, Neidenburgerstr. 43, 45897 Gelsenkirchen, Germany; dragos.pascal88@gmail.com; 3 Department of Materials Science and Testing, Westphalian University of Applied Sciences Gelsenkirchen Bocholt Recklinghausen, Neidenburgerstr. 43, 45897 Gelsenkirchen, Germany

**Keywords:** WC-Co powder, NiP powder, WC-Co-NiP, vacuum brazing, functional coatings, friction and wear

## Abstract

The present study aimed to investigate the tribological behavior of high-temperature vacuum-brazed WC-Co-NiP functional coatings deposited on 16MnCr5 case hardening steel. Dry sliding wear resistance was evaluated using a non-conformal ball-on-disk arrangement, at room temperature against 100Cr6 and WC-Co static partners, respectively. Morphological, microstructural, and chemical composition analyses showed a complex, phased structure composed of tungsten carbide, nickel, and hard cobalt-based η-structure. In the testing conditions, the coefficient of friction against 100Cr6 and WC-Co counterparts entered a steady-state value after approximately 1000 m and 400 m, respectively. The wear track analysis revealed phenomena of particles trapped between the sliding bodies, as well as gradual removal of asperities. The calculations of the wear rates proved that the values were strongly influenced by properties of the sliding system, such as crystal structure, stress discontinuities, hardness, and material homogeneity.

## 1. Introduction

Friction and wear of components can be controlled and partially prevented by means of films or coatings [1,2,3]. Friction and wear are not purely phenomena that can be solely solved by simply selecting a harder material [4,5]. They emerge from the contact interaction between moving parts that is influenced by many factors, such as contact, load, speed [6,7,8], surface and base material hardness, elasticity, toughness, chemical reactions [9], and lubrication [10], and also the dominant environmental factors, including temperature, humidity, impurities, and radiation [3,11,12,13,14,15,16]. Some deficiencies causing friction and wear can be addressed rather easily, while others are more complicated to solve. The lifetime of consumer products can be extended by means of optimal material selection and wear-resistant coatings [17,18,19,20]. Friction and wear in factory conveyors, mineral processing, mixers, extruders, blowers, pumps, and regulating valves can be reduced by efficient lubrication, anti-friction or low-friction coatings, and the correct selection of materials [21,22,23,24,25,26,27]. There is a continuous aim to develop coatings with superior mechanical properties to combat wear loss [28,29,30,31]. Cermets are generally used in dry sliding wear conditions because of their relatively high hardness and wear resistance [32]. As mentioned, hardness is very often not enough to mitigate the wear phenomena but is often accepted as one of the most important indicators for wear resistance [33]. Pirso et al. successfully demonstrated that the volumetric wear of Cr_3_C_2_-based coatings decreases proportionally with the increase WC-Co and TiC-NiMo cermets’ content [34]. The WC–Co cermet is also known for its wear resistance–strength combination at temperatures up to 600 °C [35], fracture resistance, and high hardness [36,37,38]. Nevertheless, it is important to keep the Co content low in the alloy, as Fischer demonstrated, in order to provide a lower wear rate [33]. Besides the material properties, the testing environment of the reinforced particles is highly important. Kirshna et al. recently reported that, for reinforced AL7075 metal matrix composites, the wear rate decreased with the increase of the sliding speed and increased with the increase of applied load [39]. Although heavily studied, WC-Co particles continue to fascinate the research community, being recently found also as matrix material in carbon nanotubes [40] or in TiC-based hot-pressed composites [41]. The cermet particles are commonly incorporated in a matrix material, being very often deposited through various thermal spraying techniques such as plasma [40,41], cold gas [42,43,44], high-velocity oxy-fuel [35,45,46,47], air-fuel [48,49,50], spraying, or even by a process combining the last two [51].

Even though the brazing process is conventionally utilized as a joining method, the present work aimed to assess the feasibility of using this process as a coating technique for manufacturing WC-Co-NiP functional composite coatings. This technology is insufficiently studied since similar coatings are mostly manufactured through other methods such as electrodeposition, thermal spraying [52,53,54], or electro-spark deposition [55]. The present research aimed to be an extension of a previous study, in the direction of wear resistance assessment for WC-Co-NiP functional coatings, with earlier results being reported in regard to the corrosion behavior [56]. The obtained coatings are planned to be used in plastic extruded parts, more specifically, in turning screws and barrels. Corroborating information concerning microstructure, phase composition, wear, and interacting bodies’ analysis, this work presents a comprehensive view on the tribological behavior of vacuum-brazed WC-Co-NiP coatings.

## 2. Materials and Methods

### 2.1. Materials and Manufacturing

Manufacturing the currently employed polymer-bonded hardfacing tapes involved mixing 75 wt.% WC-Co, (recycled cermet powder comprised of 7.5 wt.% Co, 5.7 wt.% C, and W, as balance) with 25 wt.% NiP (brazing filler metal powder consisting of ≃11 wt.% P, max. 0.06 wt.% C, and Ni as balance); both materials are produced by the company Höganäs AB, Höganäs, Sweden. An additional 2 wt.% water-based, non-toxic fugitive organic binder was added and mixed together until the adhesive fibrillated and anchored the powder particles, allowing further processing steps. The mixture was subsequently rolled with a DRM 150 RE, Durston, UK, rolling mill to a predetermined thickness, forming a flexible cloth. The flexible cloth was laid on a 16MnCr5 hardening steel used as substrate due to its large availability and importance as structural material. The high-temperature vacuum brazing process was carried out in a cold-wall vertical vacuum furnace HITERM 80 200, HITEC Materials, Karlsruhe, Germany, at a stable pressure of around 3.0 × 10^−4^ mbar and a brazing temperature of 1000 °C, for a soaking time of 30 min. The detailed procedure of producing hardfacings with a similar chemical composition was presented in a different study [57].

### 2.2. Characterisation

**Figure 1:**
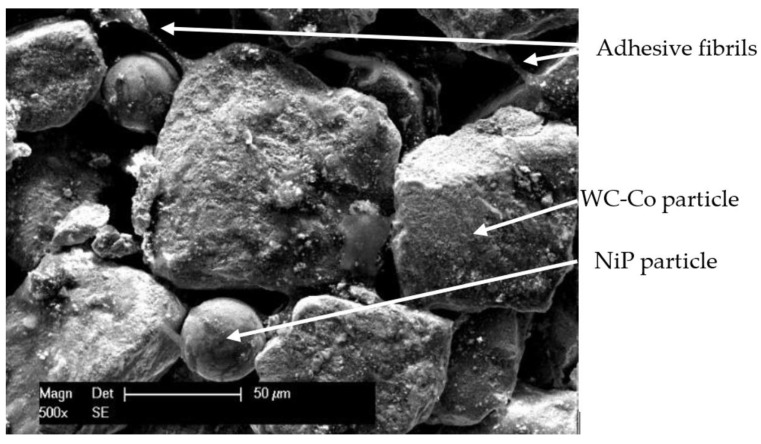
SE micrograph of WC-Co-NiP tape.

The scanning electron microscope (SEM, Philips XL, 30, Eindhoven, The Netherlands) micrograph shown in Figure 1 presents the structure of the flexible tape comprised of WC-Co powder with an irregular shape of approximately 100 μm NiP spheroidal alloy powder bound together by the organic adhesive fibrils. The micrographs were acquired with the SEM at a 10 mm working distance, at a cathode voltage of 25 kV, while the chemical composition was assessed using the energy dispersive X-ray spectroscope (EDAX AMETEK, Mahwah, NJ, USA).

The phase composition of the WC-Co-NiP composite coatings was analyzed using an X’Pert r-Ray diffractometer, Philips, Eindhoven, The Netherlands. The measurements were performed in the reflection mode, with a CuKα cathode as radiation source, at 40 kV and 40 mA, an angular resolution of 0.005° 2θ, and a scan rate of 0.01° 2θ min^−1^.

**Figure 2:**
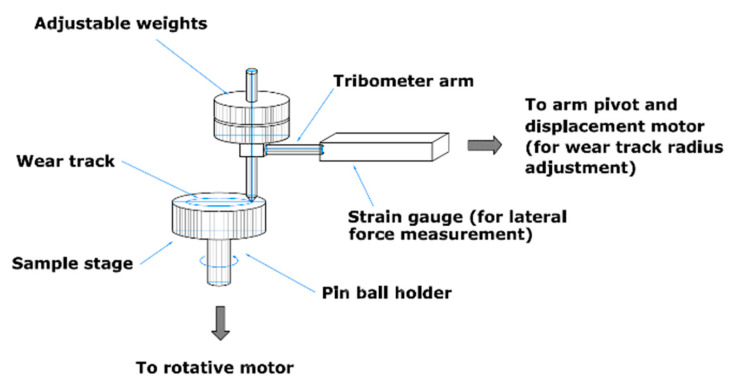
Schematics of the pin-on-disk setup.

Sliding friction and wear investigations of the WC-Co-NiP composite coatings as well as of 16MnCr5 steel used as substrate (reference sample) were performed with a CSM Instruments Tribometer, Needham Heights, MA, USA, under a non-conformal ball-on-disk arrangement, shown in Figure 2, according to DIN 50,324, with the parameters described in Table 1. The stop condition of 100,000 laps was selected to ensure that the coatings reach a steady state, ensuring that the coefficient of friction would not present fluctuations. The selected wear parameters are also typical for dry sliding pin-on-disk testing [58].

The wear tracks and the worn caps of the counter bodies were analyzed with a VK-X260K confocal laser and a VHX-600 digital microscope, both from the company Keyence, Osaka, Japan.

## 3. Results and Discussions

**Figure 3:**
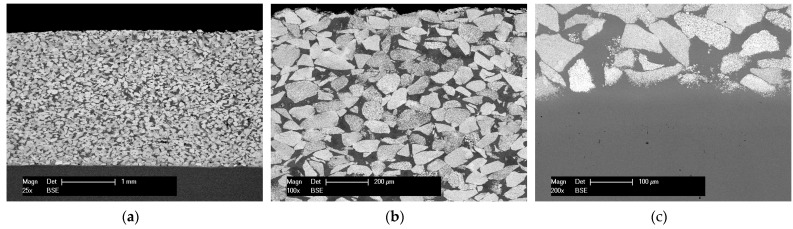
BSE micrograph (**a**) overview, (**b**) detail, and (**c**) interface with the substrate of the WC-Co-NiP coating on the 16MnCr5 substrate.

The morphology and microstructure of the coating and coating–substrate interface are illustrated in the BSE cross-section micrographs in Figure 3. The high-temperature vacuum-brazed coatings displayed a dense structure, with uniform distribution of the cermet particles. One can observe that the molten NiP self-fluxing alloy infiltrated between the WC-Co particles, cementing them together, and, at the same time, bonding the entire system with the metallic substrate. In Figure 3b,c it can be also seen that the chosen brazing temperature provided a sufficient time for the brazing filler alloy to melt, gaining the necessary fluidity to infiltrate and fill the hollow spaces between the WC-Co. The maximum temperature at which the materials were exposed during the brazing process was insufficient to melt the tungsten carbide. Nevertheless, diffusion from the metallic matrix towards the cemented WC and substrate and a dissolution of Co into the matrix was observed.

**Figure 4:**
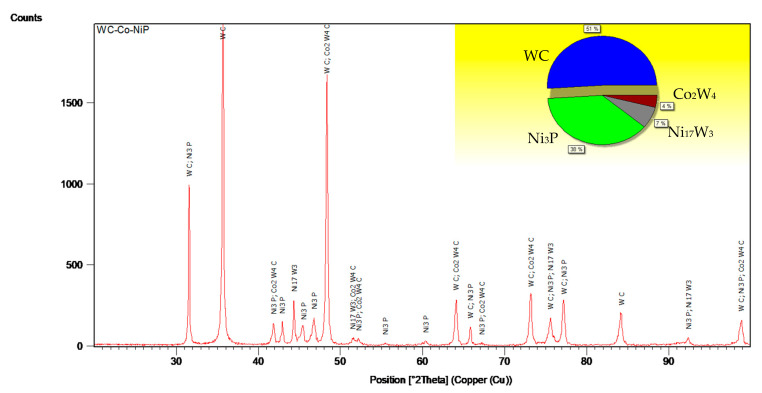
XRD pattern of WC-Co-NiP coating.

As indicated in the X-ray diffractogram (Figure 4) fitted according to the Rietvield analysis, the currently investigated WC-Co-NiP coatings were comprised mainly of 51 wt.% WC, 38 wt.% Ni_3_P, 7 wt.% Ni_17_W,_3_ and 4 wt.% Co_2_W_4_C. Considering that the Co_2_W_4_C η-phase is generally structured in small grains, Fischer demonstrated that a low Co content will reflect in a higher resistance of the material against wear [33], although previous research showed that the Co_2_W_4_C ternary η-phase is a brittle structure [59,60,61]. Due to the relatively low process temperature (1000 °C), compared to the decomposition temperature of tungsten carbide (≃2850 °C) and the lack of oxidizing elements, no decarburization of WC (normally resulting in the formation of highly brittle W_2_C or W_3_C phase) was observed. Moreover, no thermal decomposition of WC during the deposition process, in comparison to HVOF technology, was achieved [62].

Tribological characterization offers important information regarding friction, wear, and lubrication of materials under defined conditions. The wear of WC–Co-based composite coatings is generally considered to be a function of the WC particle size, WC content, and bonding strength of the WC particle with the metallic matrix. The areas of the worn track cross-section and worn cap diameter, determined by microscopic measurements, were used to calculate the specific wear applying the *Archard Equation* [63]. The rotating specimens’ worn volumes *W_v_* (mm^3^) were calculated as:

(1) $$W_{v}=2\pi r\times \frac{h}{6\times s}\left(3\cdot h^{2}+4\times s^{2}\right)$$

where *h* is the average depth (µm) and *s* is the average width (µm) of the circular segment. Accordingly, the volumetric wear rate *k* is defined as:

(2) $$k=\frac{W_{v}}{F_{N\;}\cdot s}$$

where the wear volume *W_v_* (mm^3^) is divided by the normal load *F_N_* (N) and sliding distance *s* (m), usually expressed in mm^3^ N^−1^ m^−1^.

**Figure 5:**
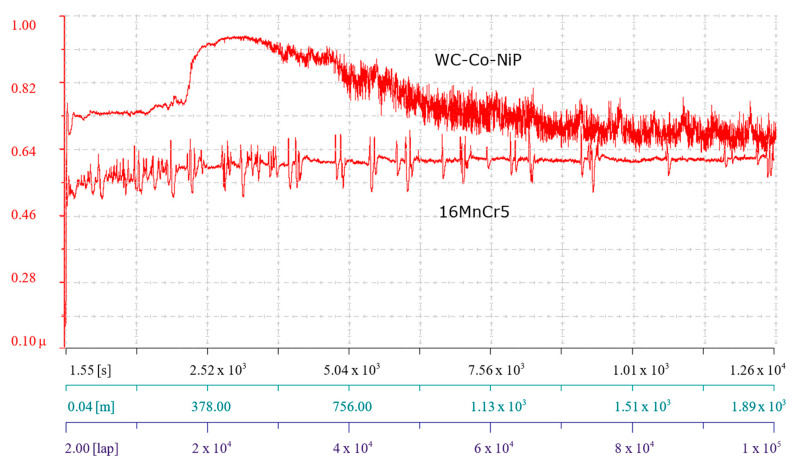
COF evolution for the 16MnCr5 substrate and the WC-Co-NiP coating vs. the 100Cr6 counterpart.

Prior to the pin-on-disk investigations performed with the parameters presented in Table 1, all samples were ground with metallographic paper to remove potential oxides and to ensure as close as possible a *Ra* 0.03 surface roughness. Before the tests, the samples were ultrasonically cleaned in acetone to remove fine particle contaminants and/or lubricants. The coefficient of friction (COF, often symbolized *µ*) was monitored and registered during the entire testing period and can be observed in Figure 5, with x axis specifying the registered time, the distance, and the number of covered laps, and the y axis, the value of *µ*.

The COF value for both types of samples (WC-Co-NiP and 16MnCr5) observed shortly after starting the investigation had a low value. However, it quickly increased, up to approximately 0.55 in the case of the substrate material and around 0.75 for the coated sample. The abrupt increase of COF was attributed to the increase in contact area between the sample and the counterbody, due to the surface roughness modification and the asperities on the sample surface. Simultaneous with the completion of the first couple of laps, a decrease was observed; however, this event lasted only an extremely short period. During the next ≈1000 laps, the coefficient of friction remained relatively stable for both samples. After an additional ≈500 laps, the WC-Co-NiP coating showed an increase in friction, up to the maximal coefficient of around 0.9, due to formation of wear particles locked between the sliding surfaces. In the same region, the COF for the 16MnCr5 reached the mean value of 0.6 and entered the steady state, which lasted until the end of the test. After the short rise in the friction value, the coating underwent a considerable decrease in COF, which subsequently stabilized at around 0.70. This reduction in frictional force was attributed to the gradual removal of asperities from the hard surface by the softer static partner. Wear particles could not, therefore, anchor anymore due to the polished surface. The numerical values of the minimum, maximum, and mean COF are given in Table 2.

**Figure 6:**
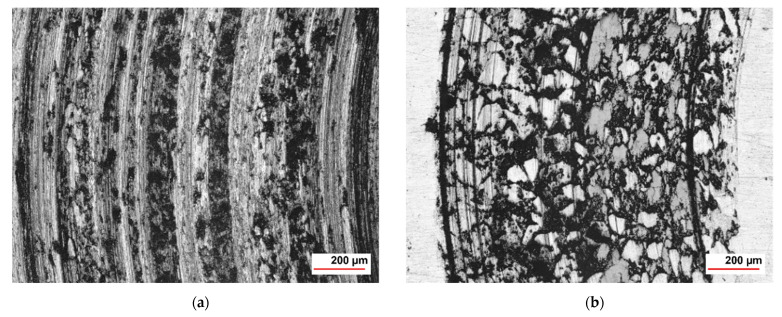
CLS micrographs of the sliding wear tracks on (**a**) 16MnCr5 and (**b**) WC-Co-NiP vs. 100Cr6.

The wear rates assessed after measuring the depth and width of the tracks left behind by the WC-Co static partner were in good agreement with the materials’ hardness value of 1010 HV10. The coating cross-section hardness values ranged from a minimum of 647 HV1 for the metallic matrix up to a maximum of 1132 HV1 of the cermet particles, respectively. The wear tracks on 16MnCr5 substrate and WC-Co-NiP functional coating after almost 2000 m and 10 N load are presented in Figure 6.

**Figure 7:**
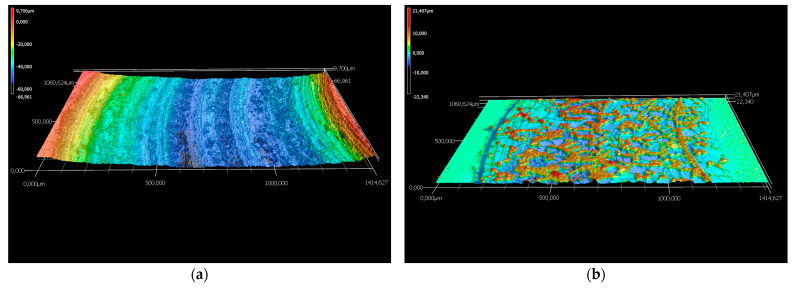
Wear track profile of (**a**) 16MnCr5 and (**b**) WC-Co-NiP vs. 100Cr6.

The considerably wider and deeper worn track found on the surface of the 16MnCr5 base metal concluded in numerical values for the wear rate of ≈ 9.1 × 10^−2^ mm^3^ N ^1^ m^−1^, compared to only ≈ 5.4 × 10^−4^ mm^3^ N ^1^ m^−1^ in the case of the composite hardfacing. Digital micrographs of the coated surface (Figure 6) display areas where the pull-out caused by friction welding (adhesion) between the coating material and the relatively soft static partner occurred. The presence of adhesion is also denoted by the frequent fluctuations of COF during the second steady-state region. Furthermore, the formation of an unstable iron oxide film and galling caused additional friction and adhesion between the static partner and the sliding WC-Co-NiP specimen, as presented in Figure 7.

**Figure 8:**
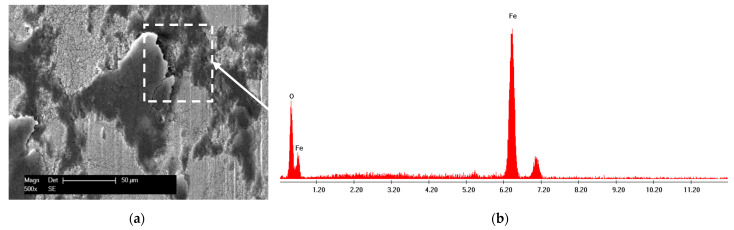
SEM micrograph of (**a**) wear debris and (**b**) corresponding EDX spectrum of WC-Co-NiP vs. 100Cr6.

In the case of sliding arrangements involving 100Cr6 against coating, soft asperities from the two surfaces in contact (metallic matrix for WC-Co-NiP coating) adhered strongly to each another, resulting in junctions and material transfer. Subsequently, the auto-lubricating tribo-layer was unstable and repetitively destroyed, the reason for the high coefficient of friction for the WC-Co-NiP to 100Cr6 combination. The fine plugging out of materials and subsequent ploughing led to the formation of grooves. The mechanism of groove formation involved ploughing of the soft surface by the hard WC particles [14]. Even though the wear track for the coated sample was relatively wide, it lacked depth and, thus, it was only superficial. This feature was attributed to the fact that material removal took place mostly at the softer 100Cr6 ball. The SEM micrograph and EDX spectrum of WC-Co-NiP coating against 100Cr6 static partner confirmed the previous affirmations, displaying in Figure 8 the presence of iron oxide scales onto the surface of the wear track.

**Figure 9:**
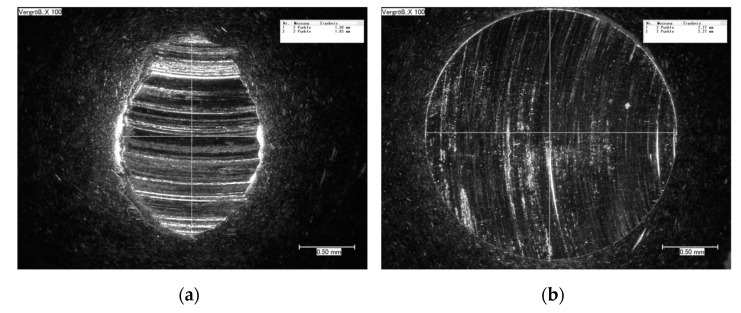
Digital micrographs of the 100Cr6 static partner against (**a**) 16MnCr and (**b**) WC-Co-NiP.

Moreover, the 100Cr6 static partner wear rate concluded in numerical values of around 5.3 × 10^−6^ mm^3^ N^−1^ m^−1^ against 16MnCr5 and a significantly higher value of approximately 2.4 × 10^−5^ mm^3^ N^−1^ m^−1^ against WC-Co-NiP. The substantial difference is clearly noticeable in the digital micrographs corresponding to the worn caps from the 100Cr6 static partner illustrated in Figure 9.

In the case of dry sliding against the hard WC-Co-NiP counterface, the specific wear coefficient of the softer 100Cr6 steel was relatively high. In the case of couplings between similar hard materials, the wear coefficient was expected to be lower. Therefore, by a proper selection of a tribological pair taking into account parameters such as elastic modulus, thermal expansion coefficient, stress discontinuities, or crystal structure, it was possible to achieve lower wear rates [3].

**Figure 10:**
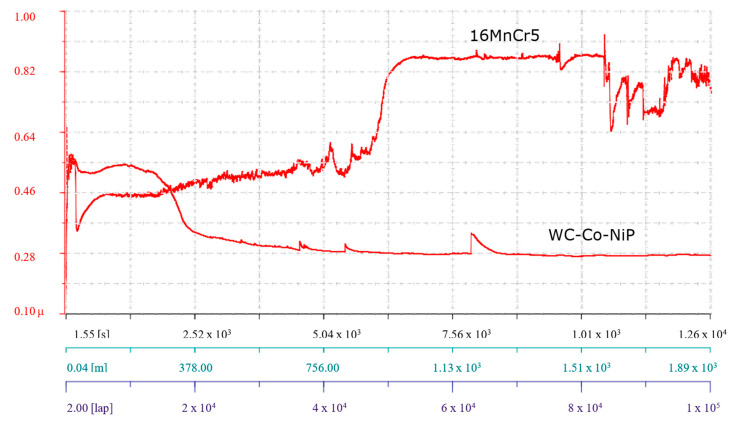
COF evolution for the 16MnCr5 substrate, the WC-Co-NiP coating vs. the WC-Co counterpart.

Because friction is not merely a material property but a system response, tribological POD investigations were also performed against a WC-Co ball, maintaining the rest of the testing parameters identically, as in the previous case. Accordingly, the coefficient of friction was once more monitored and registered during the entire testing period and can be observed in Figure 10, with the *x* axis specifying the registered time, the distance, and the number of covered laps, and on the *y* axis, the value of *µ*.

The friction development followed a chronological evolution, with a strong influence on the frictional behavior. Basically, both tested systems started with a run-in stage followed by a steady-state stage, which the WC-Co-NiP coating maintained until the end of the test, and ended in breakdown in the case of the unprotected 16MnCr5 base metal.

The lowest value for both types of samples coincided with the start of the investigation. However, it quickly changed when the systems entered kinetic friction, increasing up to approximately 0.55 for both materials. Concomitant with the completion of the first couple of laps, a decrease was observed. This phenomenon lasting only a very short period was due to a reduction in surface roughness, being more visible for the substrate material because of the lower bulk hardness (substrate hardness of approximately 550 HV1). Subsequently, after ≈1500 laps, the coefficient of friction rose again for the base metal during the next ≈50000 laps. Contrarily, after ≈1000 laps, the WC-Co-NiP coating showed a lessening in friction, which reached steady state after ≈40,000 lap and remained generally stable, with a coefficient of around 0.28 until the end of the test. Friction reached quickly a steady-state value because the coating surface was easily polished by the hard, static partner (WC-Co ball) and developed a tribo-layer. When the COF for the uncoated 16MnCr5 sample reached approximately 0.46 (after ≈1000 laps), it also entered steady state. This lasted up until the surface degraded and the friction became highly unstable, a behavior that is accordant with severe wear. In the meantime, the hardfacing showed only a few deviations, attributed to sporadic pull out of the metallic matrix caused by adhesion (friction welding) [64]. The transferred material from the softer 100Cr6 ball to the hard coating surface hindered the self-lubricating nature of the WC-Co-NiP overlay, normally provided by the Ni_3_P phase.

Decisively, a maximal friction coefficient of 0.76 was measured during the ball-on-disk testing of the 16MnCr5 steel substrate, with a mean of 0.50. Significantly lower values were determined in the case of the WC-Co-NiP functional coating, registering a maximum of 0.57 with an average of 0.33, as displayed in Table 3.

**Figure 11:**
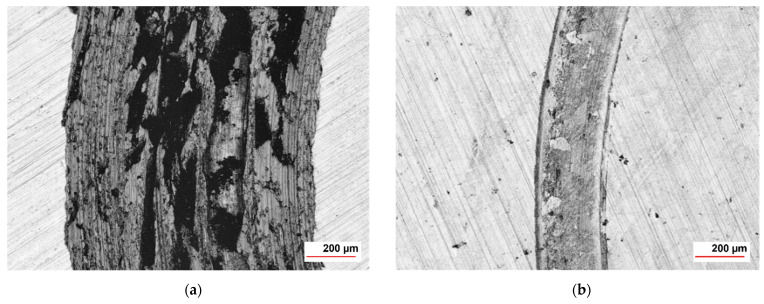
CLS micrographs of the sliding wear track on (**a**) 16MnCr5 and (**b**) WC-Co-NiP vs. WC-Co.

The wear rates assessed after measuring the depth and width of the tracks left behind by the WC-Co static partner were in good agreement with the results of the previous investigations. One can clearly notice the difference in sliding wear behavior between 16MnCr5 substrate and WC-Co-NiP functional coating in the micrographs of Figure 11.

**Figure 12:**
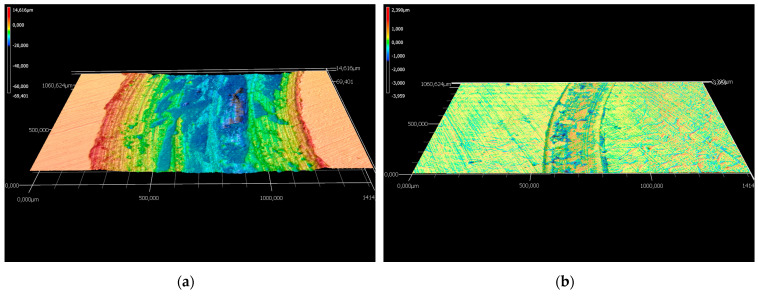
Wear track profile of (**a**) 16MnCr5 and (**b**) WC-Co-NiP vs. WC-Co.

The considerably wider and deeper worn section found on the 16MnCr5 metallic surface concluded in numerical values for the wear rate of 1.0 × 10^−3^ mm^3^ N^−1^ m^−1^, compared to only 1.5 × 10^−4^ mm^3^ N^−1^ m^−1^ in the case of the WC-Co-NiP composite hardfacing. Furthermore, the formation of an unstable iron oxide film and galling, causing additional friction and adhesion between the static partner and the sliding (rotating) 16MnCr5 case hardening steel specimen, can be observed in Figure 12a. The digital micrograph from Figure 12b of the coated surface wear track displays sporadic areas where the pull-out occurred.

**Figure 13:**
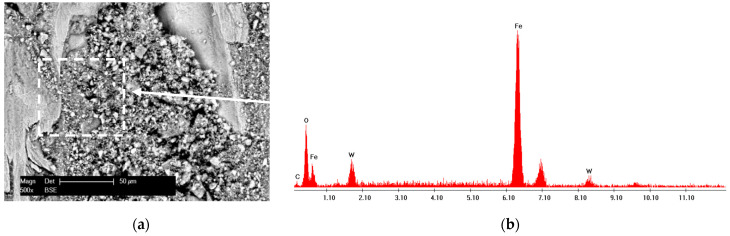
BSE micrograph (**a**) and corresponding EDX spectrum (**b**) from the wear track of 16MnCr5 vs. WC-Co.

The SEM micrograph and EDX spectrum corresponding to the wear track of 16MnCr5 steel against the WC-Co static partner confirmed the formation of an unstable iron oxide layer. Additionally, the transferred material comprised of WC particles from the WC-Co ball was found embedded in the wear track of the 16MnCr5 steel sample, as illustrated by Figure 13.

As already mentioned, wear caused by severe adhesion between the sliding surfaces in the form of galling was observed on uncoated samples in both testing conditions, specifically versus 100Cr6 as well as WC-Co static partners. Galling is a type of surface damage manifesting between sliding objects, distinguished by typically localized macroscopic roughening, and formation of protrusions above the initial surface; it frequently involves plastic deformation and/or material transfer [65]. It occurs under transverse motion and especially if the tribo-system is not or is poorly lubricated. According to J. R. Davis [66], the propensity of a material to gall is affected by its ductility; thus, harder materials are more impervious to galling. Correspondingly, plastic deformation behavior plays a key role in severe adhesive wear. Materials with a face-centered cubic lattice are exceedingly prone to galling due to effortless cross slip of dislocations. Alloys or elements that crystalize in systems with lower stacking-fault energy (i.e., body-centered cubic structure) are less susceptible to galling. To an even greater degree, hexagonal, closely packed materials are extremely resistant to galling [66]. The damage on the uncoated samples showed characteristic galling patterns, with an uneven surface, caused by severe plastic deformation. Moreover, large volumes of material are adhesively transferred, indicating the perpetual destruction of the surface oxide tribo-layer. This was more easily noticeable for the 16MnCr5 versus WC-Co, where, in the vicinity of the worn caps, transfer material is present. On the contrary, due to the high amount of WC with an HCP lattice and Ni_3_P crystallized in the body-centered tetragonal (BCT) system, the coated samples showed little to no signs of severe adhesive degradation. Contrasting other types of wear, galling happens rapidly and is typically not a progressive process. Accordingly, it is safe to assume that the WC-Co-NiP coatings will not suffer wear by galling even under prolonged exposure to the testing conditions employed in the current tribological investigations.

**Figure 14:**
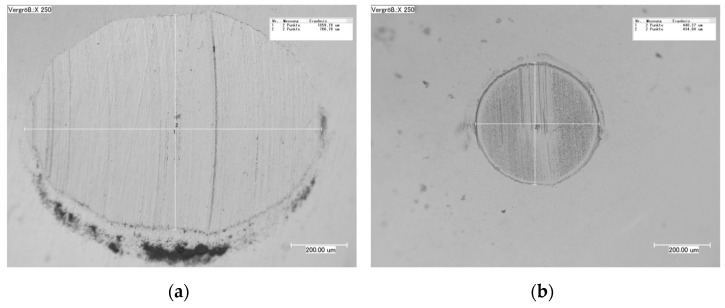
Digital micrograph of WC-Co static partner against (**a**) 16MnCr5 and (**b**) WC-Co-NiP.

Furthermore, the WC-Co static partner wear rate concluded in numerical values of 6.0 × 10^−7^ mm^3^ N ^1^ m^−1^ against 16MnCr5 and a considerably lower value of only 3.2 × 10^−8^ mm^3^ N ^1^ m^−1^ against WC-Co-NiP. This significant difference is clearly noticeable in the digital micrographs corresponding to the worn caps from the WC-Co static partner displayed in Figure 14.

The main mechanisms that govern the surface degradation in the currently investigated sliding systems were adhesion and tribo-oxidation. If the tribological system comprises third bodies, namely, hard particles (much harder than the two surfaces in contact), abrasive wear might also occur [67]. This was found to be the case of the coupling between 16MnCr5 and WC-Co, which led to the formation of abradable WC particles. Thus, the wear mechanism changed from an adhesive and tribo-oxidative one to a process that also encompassed abrasion.

In the case of dry sliding against the 16MnCr5 steel counterface, the specific wear coefficient, of the WC-Co static partner was quite low. In the case of couplings with the similarly hard WC-Co-NiP material, it was even lower. Therefore, as already explained, the optimal selection of a tribological pair led to very low wear rates for both tribo-partners.

## 4. Conclusions

Successfully brazed WC-Co-NiP samples deposited on a 16MnCr5 structural steel substrate were obtained at 1000 °C in a vacuum furnace at a pressure atmosphere of 3.0 × 10^−4^ mbar. The microstructural characterization showed a dense structure with a uniform cermet particle distribution bonded together with the metallic substrate. The diffractometric analysis combined with Rietvield calculations revealed that the phases were 51 wt.% WC, 38 wt.% Ni_3_P, 7 wt.% Ni_17_W_3,_ and 4 wt.% of the brittle Co_2_W_4_C η-structure. Due to the relatively low process temperature, no decarburization of the cermet phase could be observed.

Non-conformal pin-on-disk coefficient measurements against steel and tungsten carbide counter bodies revealed phenomena of wear due to particles locked between the sliding surface, gradual removal of asperities from the surface, or removal of the unstable auto-lubricating tribo-layer. Nevertheless, no evident correlation between the coefficient of friction and the wear rate could be established. The wear rate calculation using the Archard equation unveiled that, against the 100Cr6 ball, the WC-Co-NiP showed a higher wear rate than the steel substrate. This evidences that the selection of a proper tribological pair needs to be made considering parameters such as the thermal expansion, stress discontinuities, or crystal structure.

Looking at the tests against the much harder WC-Co counter body, a much lower wear rate of the developed WC-Co-NiP coating was seen. WC particles transferred on the WC-Co-NiP were found embedded on the wear track. Wear caused by severe adhesion between the sliding surfaces in the form of galling, causing plastic deformation, was observed on both samples’ surfaces. A much smaller worn cap area of the WC-Co counter body on the WC-Co-NiP was as well detected in comparison to the 16MnCr5 sample. Due to the similar material properties in the sliding tests between the WC-Co static partner and the 100Cr6 substrate that did not comprise third bodies, abrasive wear also occurred. The promising results conclude that the coatings can be potentially employed as wear-resistant materials for plastic extruded parts such as turning screws and barrels.

## Figures and Tables

**Table 1:** Pin-on-disk test parameters.

Static Counterpart	Radius (mm)	Linear Speed (cm s^−1^)	Normal Load (N)	Laps	Total Distance (m)	Test Duration (s)
WC-Co ball	3	15	10	100,000	1890	12,600
100Cr6 ball	3	15	10	100,000	1890	12,600

**Table 2:** Coefficients of friction and wear rates for the POD tests vs. 100Cr6 static partner.

Sample	COF Min.	COF Max.	COF Mean	Wear Rate (mm^3^ N^−1^ m^−1^)
16MnCr5	0.17	0.70	0.60	9.1 × 10^−2^
WC-Co-NiP	0.16	0.95	0.77	5.4 × 10^−4^

**Table 3:** Coefficients of friction and wear rates for the POD tests vs. WC-Co static partner.

Sample	COF Min.	COF Max.	COF Mean	Wear Rate (mm^3^ N^−1^ m^−1^)
16MnCr5	0.18	0.76	0.50	1.03 × 10^−3^
WC-Co-NiP	0.21	0.57	0.33	1.55 × 10^−4^

## Data Availability

The data in this study is available on request from the authors. The data are not publicly available because they are part of ongoing studies.
